# Precision Feeding of Feedlot Calves Based on Phenotypic Production Profiles II. The Economic Value in a Feedlot Model

**DOI:** 10.3390/ani15131900

**Published:** 2025-06-27

**Authors:** Andreas H. R. Hentzen, Dietmar E. Holm

**Affiliations:** Department of Production Animal Studies, Faculty of Veterinary Science, University of Pretoria, Private Bag X04, Onderstepoort 0110, South Africa

**Keywords:** animal production, precision livestock feeding, phenotypic production traits, production profiling, cattle feedlot, economic returns

## Abstract

Incoming feeder calf production potential can be predicted based on phenotypic appearance, and nutrient supply can be optimized to match each animal’s specific production profile. Tailoring the supply, quality, and quantity of nutrients to the requirements for maximum profit could further support the economic pillar of sustainable livestock farming. Feeder calves (n = 104) were categorized into the production profiles (PP 1; PP 2; PP 3). Within each PP category, the allocated pens were subsequently randomized into three diets (high-, medium-, and low-production diets). Economic important traits were measured, and a deterministic model was created to evaluate economic implications. There was a significant interaction between the incoming feeder calf production profile and diet on the profit margin, with the PP 2 calves being most profitable when fed on the medium-production diet (profit margin = 4.81%). This was in stark contrast of the profit made by PP 2 calves fed on the low- or high-production diets (profit margin = 0.21% and −2.97%, respectively). PP 3 calves made a loss on all diets; however, this loss was reduced by 14% when fed on the low- compared to the medium-production diet (profit margin = −1.45% and −1.68%, respectively). PP 1 calves were profitable on all three diets although the margin was highest on the medium-production diet. In conclusion, the medium-production diet, representing the current industry norm in South Africa, is financially suitable for feeding calves with average production potential (PP 2), whereas the loss made by calves with low production potential (PP 3) can be reduced by adjusting the feed formulation for low production. More work is required to formulate diets that will maximize the profit made by calves with above average production potential (PP 1).

## Simple Summary

We previously demonstrated the ability to predict feeder calf production based on visual phenotypic classification of production profile (PP), and how this can be applied in a commercial feedlot to match the nutritional needs of animals through precision livestock feeding. This paper demonstrates the feasibility of implementing precision livestock feeding based on phenotypic traits (PP 2 = average, PP 3 = below average, and PP 1 = above average production potential), as measured using an economic feedlot model. The profit maximization reformulation of feedlot diets based on the production potential (PP), and therefore the nutritional needs of individual incoming feeder calves, holds the key to the future financial sustainability of cattle feedlots.

## 1. Introduction

South Africa is estimated to have a cattle population of 13.3 million, made up of 5.69 million cattle owned by 240,000 small scale farmers and about 3 million subsistence farmers, with the balance owned by approximately 22,000 commercial livestock farmers [1]. Because most feedlots have some commercial interest in a particular abattoir, the feedlot industry is primarily responsible for the increased vertical integration of the red meat value chain and the beef supply chain [2]. Carcass auctions at the major urban abattoirs were used to determine prices in the formal markets prior to the deregulation of South Africa’s red meat sector [3]. Since deregulation, market forces based on supply and demand have shaped prices [3]. Historically in South Africa, feeder calves typically arrived in the feedlot weighing between 200 and 220 kg and remained for approximately 100 days on feed [1], during which time they acquired an additional 100 kg, bringing their final carcass weight to between 220 and 225 kg [1]. In 2017, the approximate feedlot entry weight was 253 kg, with an exit weight after a feeding period of 135 days of approximately 465 kg resulting in a carcass weight of around 272 kg [4]. A number of factors, including improved management, nutrition, the use of growth enhancers and beta-agonists, management, and consumer demand outpacing supply, have contributed to the increase in beef feedlot productivity during the past 20 years [4,5,6,7,8]. Beef production in South Africa, however, remains under pressure due to various factors, such as biosecurity breaks and disease outbreaks, fluctuating feed costs, high interest rates, and inflation affecting consumer buying power [9]. The per capita domestic consumption of beef in South Africa declined over the past two decades, resulting in the country becoming a net exporter of beef, with the net export of beef being projected to grow from around 4% to around 7% of total production in the next decade. This contributes to a positive outlook for the South African beef industry in the medium term [9].

Short term variation in profitability of beef feedlots is largely caused by variation in feed costs and the price of feeder calves [10]. In South Africa intensive feedlots provide the biggest market for primary producers to sell their weaners to. It is estimated that 75–85% of the total weaners produced in South Africa are taken up by feedlots [4]. Many factors influence the performance and profitability of an individual feedlot calf [11,12,13]. Therefore, for beef feedlots to maintain their profitability, new methods and strategies to increase production efficiency must be sought, specifically by reducing the cost of gain per individual calf [9,14,15]. Feedlots add value to the feeder calf by converting feed into carcass. Feedlots attempt to achieve this at the best possible feed conversion ratio (FCR) and over the shortest possible feeding time (days on feed or DOF).

Quantitative measures are used to describe growth [16]. An animal’s growth can be assessed using measurable anthropometric characteristics other than weight [16]. For a long time, linear assessments of bone size have been employed to forecast an animal’s future growth performance [17]. The sorting of calves at the end of the backgrounding phase can be improved by taking direct measurements of hip height, body weight, upper arm thickness, and other factors [17]. The shortfalls of linear measurements and their execution have been described previously [17]. The production profile (PP) classification system developed for receiving feeder calves (before the feedlot phase) is a crucial addition to mitigating the financial risks for the feedlot on an individual animal basis [17]. This novel PP classification method predicts feedlot growth performance before the feedlot phase commences.

The PP classification results have shown that by predicting feeder calves’ live and carcass performance when entering the feedlot, the growth efficiency per PP class can be optimized by providing the relevant nutrients [9,18].

Price margin, feed margin, and other costs determine a feedlot’s profit [12,19]. A feedlot’s profit margin is influenced by a number of factors, including pricing margin, feed margin, management, feed cost, feeder purchase price, and selling price, which is typically expressed as carcass price [1]. The point in a feedlot where the total income per kilogram of beef sold matches the total input cost per kilogram of beef produced is known as the breakeven point [3]. Purchase price, feed cost, yard cost, and marketing costs are all considered input costs, whereas revenue costs include money from beef, offal, hide, and other sources [3,14].

The price margin (calf purchase price vs. meat price) and the feeding margin (feed costs to produce 1 kg of meat vs. the price of 1 kg of meat) are key economic margins in the feedlot [4,12]. The profit or loss that the feedlot experiences as a result of a change in the value between the time that the animal is purchased (cost price) and when it is sold (sale price) is known as the price margin [1]. The difference between the buy and sale prices, which is affected by changes in the price of beef as well as improvements in carcass quality brought about by feeding, is included in the price margin [1]. The calf price should be less than 55% [3], but it is currently closer to 60% when represented as a percentage of the beef price (calf price per kilogram of live weight supplied to the feedlot divided by the beef price achieved). It is important to note that this serves as a guideline only. The weaners’ purchase price is influenced by supply and demand, but it also depends on global meat trends and the current and anticipated grain prices [3]. The feedlot industry is a high-risk enterprise, where the PP classification and feeding to the production group can add value. It is significant to note that South Africa is the only country in the world where the price of the final carcass is unknown when the weaner is purchased [1]. The most desired economic situation for a beef feedlot is where cattle diets are formulated to a maximum profit (in contrast to least cost or maximum weight gain diet formulation). A mathematical model for the profit-maximizing of cattle diets was developed, demonstrating the differences between net energy requirements to achieve maximum profit, maximum growth or least-cost, in the formulation [20]. In fact, the net energy required for maximum profit formulation appears to lie somewhere between that for least-cost and that for maximum growth [20]. Their study, like other similar studies investigating the efficiency of diet formulation systems, was based on the group average, which represents animals with varying production potential within the group. Such models as this can be used as a basis for developing profit-maximizing diets that are best suited for the predetermined growth potential of subsets of animals within a feeder calf population using production profiling, or even on an individual animal basis in future [20].

This study investigates whether the subsequent precision feeding unlocks the economic value of the PP classes by optimizing the carcass feed cost of gain (CFCOG) per PP classification, which might then lead to better margins over feed cost and improved economic viability of beef feedlots. The objective of the study is to determine if mitigating procurement strategies can influence the value and cost of carcass gain through precision feeding.

## 2. Materials and Methods

The experimental design was described previously [21,22,23]. Feeder calves were categorized into the four production profiles (PP 1; PP 2+; PP 2−; PP 3) after being weighed and individually identified at the original commercial feedlot [16]. In brief, PP 2 represents the average feeder calf in terms of production potential based on phenotypic appearance (muscling), with PP 2+ and PP 2− being those calves above and below the average, respectively. PP 3 represents calves with the lowest production potential, based on a lack of muscling and bone structure to achieve optimal carcass development. PP 1, on the other hand, represents calves with the highest production potential: these are calves with exceptional muscling and a structural frame (bone development) that supports maximum carcass gain [17]. On arrival at the experimental farm, each of the 4 PP classifications consisted of 27 animals that were randomly allocated into 36 pens in total after ranking based on weight. There were three pens for solitary animals and six pens with four inmates each for each PP category [21]. Using a random number created in Microsoft Excel (MS Office), the allotted pens within each PP category were then divided into three diets: a low-production diet (LPD: 13%CP; 10.6 MJ/kg ME), a medium-production diet representing the norm in South African beef feedlots (MPD: 14%CP; 11.3 MJ/kg ME), and a high-production diet (HPD: 15%CP; 12.0 MJ/kg ME) [21]. After 142 days of feeding, feeder calves were slaughtered.

Prior to analysis, data of PP 2+ and PP 2− were combined in order to represent the entire cohort of animals that are considered to represent the average feeder calf, following which, animal production data (Table 1) were used to enter into a deterministic break-even model of beef feedlot profitability, Oklahoma State University [19]. The above model was adapted to determine cost on a carcass basis, rather than live weight, since beef is traded on a carcass basis and not on a live weight basis [21]. This was achieved using the methodology described by us previously [24], whereby live entry-weight is converted to carcass weight at feedlot entry using the following formula: CW = 0.694 × SBW − 38.43 kg, where CW = calculated carcass weight at feedlot entry and SBW = shrunk body weight at feedlot entry [24]. Following this conversion of the live entry weight to the carcass weight at feedlot entry, all other relevant parameters in the Oklahoma State University feedlot profitability model were adjusted to a carcass basis [19,24].

The value of precision feeding calves to their potential (based on PP classification) and the potential economic impact is further investigated using the above-mentioned deterministic brake-even model. Detailed economical modeling of beef feedlots has been described [14]; however, in this study, all other variable costs (sanitary management, identification management, taxes), and all semi-fixed-, fixed- and remuneration costs were the same for the different study cohorts; therefore, only the feed costs and animal purchase costs were modeled. The combination of feeder calf purchase cost and feed costs has been reported to represent about 85% to 90% of total costs [4,14]. The calf purchase cost was calculated on a carcass basis [21] using the same South African Rand (R)-value per kg for all animals (R57.12/kg), plus 10% interest over the feeding period (Table 2). The intake (kg feed) multiplied by the monetary value of the diet (R/kg) over the 142-day feeding period was calculated to represent the feed cost (Table 2). The feed costs included in the model were the actual costs incurred by the experimental facility and were higher than the industry norm (Table 1). Interest on 50% of the feed costs was added to determine the final feed costs, as is the practice of cattle feedlot farmers because of frequent feed supply purchasing [14]. The carcass income was the value of the carcass, which was calculated as the carcass exit weight (kg) multiplied by the carcass price (R55.00/kg for all carcasses) (Table 2).

## 3. Results

This research demonstrates the value of the improved production parameters of the PP 1 calf (Table 1). Feeding the production profiled (PP) feeder calf to its potential has further increased the value in terms of income per carcass. The PP 1 calf had the highest income on all three diets; however, the profit of the PP 1 calf fed on HPD was lower due to a 13.74% higher feed cost. The PP 3 feeder calves fed the LPD had the lowest total cost and the lowest total income, resulting in an overall loss.

This research further demonstrated the value, both in monetary terms and in policy terms, of feeding the PP 3 calf on a LPD. The PP 3 calf had the smallest financial loss when fed the LPD, a 14% improvement in profit margin over the PP 3 calf fed the current feedlot norm (MPD).

The entry weight of the feeder calves differed between PP classes (Table 1), and PP 1 being the heaviest resulted in PP 1 having the highest purchase cost and PP 3 having the lowest. The diet cost was the highest for HPD and the lowest for LPD (Table 1). In this study, we had the data to calculate the actual CFCOG. We looked at the feed cost incurred to produce 1 kg of carcass. The lowest CFCOG of R45.94 was seen in the PP 2 on the MPD (representing a commercial feedlot ration). Considering the respective intakes and comparing the PP within the three different diets, PP 3 had the lowest feed cost, and PP 1 had the highest, irrespective of the diet fed. Feeding the same MPD to the PP 1 feeder calves resulted in the next lowest CFCOG of R48.27.

The economically important production parameters were the best for PP 1 (Table 1) and supported PP 1’s highest income per diet fed. The higher total income was, however, not enough to realize the highest profit on a per-diet basis in the case of PP 1. The highest profit was realized by the PP 2 animals (representing the average animal) on the medium-production diet (MPD, being the average diet). The PP 3 calves had the highest profit when fed the LPD and made a loss on the HPD. The PP 1 calves realized the highest profit when fed the MPD, when comparing them on the different diets (Table 2).

## 4. Discussion

### 4.1. Precision Feeding to the Production Potential (PP Classification) in Feedlots

It is clear that in the intensive beef production system, the growth performance of cattle is of economic importance [12,25], being influenced by the plane of nutrition, hormonal status, and the environment [21,26]. Performance by feedlot animals and the importance of daily gain and dietary energetics are described and are in line with this research [24]. Maximum growth rate may not always be the most lucrative, but high growth rates are chosen because of the earlier marketing weight that is achieved as compared to slow growth [27,28].

Frame and muscle scores [21,23,29] have been shown to affect the days on feed in addition to carcass composition [29], as is evident from the additional muscle produced by especially the PP 1 class animal in this research over the same period (DOF), more effectively (higher CADG), and in a more efficient (lower CFCOG) way.

Decision-making in feedlots should consider the variation in production potential (PP) caused by the cattle’s prior environment and genetic potential [30]. The establishment of PP classification is a key mitigator for the possible production variation risk. Cattle can be sorted into homogeneous groups before entering the feedlot using direct measures of body size and condition [31] and now also PP classification. The feedlot will benefit financially as a result of groups of cattle having more uniform feed efficiencies (CFCR) and endpoint carcass features [32]. Furthermore, PP classification enables the feedlot to challenge-feed the feeder calves with better production potential (PP 1) to unlock the superior economic carcass production parameters and their value.

### 4.2. The Economic Impact of Precision Feeding Feeder Calves to Their Predetermined Production Potential (PP) Under South African Market Conditions

The feed cost of gain (maximum profit reformulation) holds the key to unlocking the value of the PP 1 calf fed on a cost-effective diet that is better suited to the animal’s superior production potential. The application of the value in procurement strategy needs further research into the price margin per PP category. It is clear that PP 1 has more value than PP 3, but for it to be incorporated into a buying strategy, more research is needed, using stochastic modeling based on more data (animal performance as well as economic data) representing more than just the current feeding scenario.

The economic model is in line with and further supports the findings of a previous study [17], i.e., that an animal with poor production profile cannot be fed to perform economically better than its inherent potential, and a better strategy seems to be to establish a diet that limits the financial losses of such an animal.

The difference between a feedlot’s profit or loss from live weight gain and the cost of feed is known as the feed margin [1]. Realizing targeted growth rates and taking action to obtain the best feed price will guarantee a positive feed margin [1]. The relationship between supply and demand determines feed pricing. Weather is the most significant of the many factors that affect supply. Feedlot operators have little influence on the feed price but can improve the efficiency of growth and the feed-carcass gain ratio, as shown in this research. Once again, part of the PP classification research showed a direct effect (PP 1 fed the HPD in Experiment 2). Repeatedly, the PP 1 outperformed the other PP classes in the measured production parameters. The degree observed is dependent on the diet fed (Table 2). It is reasonable to say that the PP 1 should positively affect the feed margin by being more efficient, i.e., by using less feed to gain 1 kg of carcass; however, this will require improved profit-maximizing reformulation of the diet to suit the superior growth potential of PP 1 feeder calves [20].

The monetary value of producing one kg of beef, carcass feed cost of gain, is a supported financial measurement that is used for feedlot decision-making [14,27,33]. The demonstration of the lowest CFCOG in the PP2 on the MPD further supports the argument that in the South African Feedlot industry, feedlot diets are optimized to meet the nutritional requirements of the average feeder calf. The finding that PP1 feeder calves have the second lowest CFCOG can potentially be explained by the fact that the growth potential of the PP 1 calves did not match the MPD, as was the case for the PP 2 calves, seeing that the PP 1 calves had higher feed intakes than the PP 2 (+0.75 kg) without achieving a higher growth rate, resulting in higher CFCOG (Table 2). The PP 3 classification feeder calves fed on the same MPD had a CFCOG of R 50.91. Fed the same MPD, this is at least R4.97 more feed cost to gain 1 kg of carcass and indicates that this is not economically sustainable. Comparing it to a commercial feedlot, the MPD is a reasonable-to-good match when fed to PP 1 and PP 2 calves, respectively, but not to the PP 3 calves.

The diet cost of the HPD that supported the PP 1 classified feeder calves in achieving better growth parameters was too high, resulting in an increased CFCOG (R49.82). The high diet costs of feeding the HPD to PP 1 in this scenario proved to be less cost-effective than feeding the MPD, as well as the LPD to the PP 1. The PP 1 feeder calf had a lower CFCOG (R48.94) on the LPD than on the HPD, despite eating less HPD and having better growth on the HPD. To unlock the relevance of the HPD further, reformulation is necessary, to use the better growth potential, but at a lower CFCOG.

Feeding the PP 3 feeder calves more cost-effectively is another point deserving our attention. The lower growth potential is one of the major factors contributing to the high CFCOG. The poorer growth potential could not be completely offset by a cheaper ration in terms of overall profit; however, the LPD being about 6% cheaper than the MPD resulted in better CFCOG for the PP 3 animals when compared to PP 3 animals fed on MPD. In addition to the lower feed cost, PP 3 had a lower intake on the LPD, contributing to the better CFCOG. Ideally, as with the HPD, reformulation for improved CFCOG could result in feeding PP 3 calves profitably.

The absence of precision feeding in the South African feedlot is a constraint to sustainable beef production. This study demonstrates the importance of determining which animal has the lowest CFCR and the greatest CADG. Feedlot economics may be enhanced by providing these individuals with the nutrients that promote CADG and CFCR. In the intensive feed sector, feeding to the production potential is relevant when the cost of those nutrients is cost-effectively matched to the relative growth potential. Precision cattle farming’s economic pillar is supported by precision feeding, which improves the value of gain and lowers CFCOG [34].

Once the most cost-effective match is established, feeder calves’ purchase price can be adapted, based on a cost-effective response to the rations that support that PP classes. For example, knowing that a PP 3 has the poorest CADG and CFCR, and thus that it influences CFCOG, is essential information for the purchase price. Precision feeding improves the value of gain, decreases CFCOG, and thus supports the economic pillar of precision livestock farming [34]. Where a feedlot feeds to the average (MPD), and as shown, PP 1 and PP 2 are good matches to that diet based on the CFCOG, PP 3 can be purchased at a lower unit price, to make up for the poorer CFCOG. This is if formulating and feeding another diet for PP 3 is no option.

The cost of purchase and feed was not low enough to offset the lower income generated from production (CADG; CFCR). Earlier observations that PP 3 feeder calves cannot be fed into profit is further supported by an increasing loss when fed the MPD and HPD. When the PP feeder calf was fed the LPD, it resulted in a reduction of 49.6% of the loss incurred compared to the PP 3 feeder calf fed the MPD, as is the norm. Although a *p*-value cannot be assigned to this, due to the magnitude of change, this is likely to be significant and repeatable in other datasets. The precision feeding of a reformulated diet resulted in a reduction in the overall loss. For the PP 3 feeder calf to become profitable, further reformulation for profit maximization is called for [20,35], which could address the feed cost of gain.

The application of classifying feeder calves into PP and subsequently feeding them to their growth potential, in a cost-effective way, is feasible in the South African Feedlots. The model is a theoretical calculation that supports and is in line with the measured economic growth parameters. The model further illustrates the possible value and, at the same time, points out the importance of weight (entry weight) at the time of purchase. This is important to note when establishing procurement strategy protocols, which need to be investigated in further studies [36]. This model should be validated by obtaining more data and using a stochastic financial model to consider variability in the input variables as well as financial/market factors.

## 5. Conclusions

The South African feedlot industry can benefit financially from precision feeding based on production profiling of feeder calves. This deterministic financial model confirmed that the current diet formulated for a commercial cattle feedlot is most profitable in the case of incoming feeder calves with an average production potential (PP 2). Calves with a better production potential (PP 1) did indeed realize better production, but this did not result in improved financial outcomes.

However, in this study, the financial loss incurred by incoming feeder calves with a low potential of production (PP 3) was limited by 14% when fed on a low-production diet, which confirms that the profit maximization reformulation of diets holds the key to the future financial sustainability of cattle feedlots.

## Figures and Tables

**Table 1:** Experimental and financial data used to populate the deterministic financial model.

Diet	Low-Production Diet (LPD)			Medium-Production Diet (MPD)			High-Production Diet (HPD)		
Production Profile (PP) classification *	3	2	1	3	2	1	3	2	1
n	9	17	8	9	16	9	9	18	9
Mean entry weight (95% CI) (kg)	199.2 (186.1–212.3)	212.7 (204.1–221.3)	224.6 (212.6–236.7)	200.0 (181.4–218.6)	211.7 (200.2–223.2)	229.4 (213.3–245.6)	205.1 (187.4–222.8)	215.8 (207.4–224.3)	225.4 (212.0–239.0)
Mean carcass weight (95% CI) (kg)	231.1 (220.7–241.5)	269.2 (255.1–283.4)	285.8 (267.6–304.0)	252.6 (232.7–272.5)	285.2 (270.0–300.5)	301.1 (286.8–315.3)	258.7 (241.2–276.2)	294.0 (280.0–306.0)	319.1 9305.1–333.1)
Mean carcass average daily gain (95% CI) (kg/d)	0.84 (0.79–0.90)	1.04 (0.96–1.13)	1.10 (1.01–1.20)	1.00 (0.92–1.06)	1.16 (1.08–1.24)	1.19 (1.10–1.27)	1.01 (0.91–1.10)	1.20 (1.13–1.28)	1.33 (1.27–1.40)
Mean carcass feed conversion ratio (95% CI) (kg/kg)	10.62 (9.69–11.55)	10.29 (9.67–10.90)	9.82 (9.75–9.89)	9.81 (9.20–10.42)	9.04 (8.63–9.45)	9.18 (8.36–10.00)	9.40 (7.34–11.49)	8.59 (8.17–9.01)	8.03 (7.53–8.54)
Mean Daily feed intake (95% CI) (kg/d)	8.69 (7.09–10.28)	10.74 (9.64–11.85)	11.19 (10.03–12.36)	9.70 (9.51–9.89)	10.28 (9.71–10.85)	11.03 (10.35–11.71)	8.81 (8.48–9.14)	10.35 (9.65–11.05)	10.69 (9.95–11.43)
Mean carcass gain (95% CI) (kg)	119.5 (111.5–127.5)	148.3 (136.8–159.8)	156.6 (143.3–169.8)	140.5 (130.6–150.3)	165.0 (153.3–176.7)	168.5 (156.7–180.3)	143.0 (129.1–156.9)	170.8 (160.4–181.3)	189.3 (179.8–198.8)
Diet cost ** (ZAR/kg)	4.73			5.10			6.10		

* Production Profile (PP) 3 represents feeder calves with low production potential based on phenotype. Production Profile (PP) 2 is a combination of PP 2+ and PP 2− data, representing all feeder calves with average production potential. Production Profile (PP) 1 represents feeder calves with high production potential based on phenotype. ** ZAR = South African Rand

**Table 2:** Results of the deterministic financial model adapted from https://extension.okstate.edu/fact-sheets/program-to-estimate-feedlot-breakeven-purchase-price-beflcalc.html (accessed on 3 March 2025) based on actual experimental data.

	Low-Production Diet			Medium-Production Diet			High-Production Diet		
Production Profile (PP) *	PP 3	PP 2	PP 1	PP 3	PP 2	PP 1	PP 3	PP 2	PP 1
A: Purchase cost ** (ZAR)	6926.17	7395.57	7809.33	6953.99	7360.80	7976.22	7131.31	7503.35	7837.14
B: Feeding cost ** (ZAR)	5951.52	7355.50	7663.69	7152.96	7580.66	8133.73	7772.01	9130.75	9430.51
C: Carcass cost per animal ** (ZAR)	12,897.56	14,775.63	15,498.61	14,130.83	14,966.77	16,137.11	14,929.28	16,664.41	17,299.15
D: Carcass income per animal ** (ZAR)	12,710.50	14,806.00	15,719.00	13,893.00	15,686.00	16,560.50	14,228.50	16,170.00	17,550.00
E: Carcass feed cost of gain ** (ZAR/kg carcass)	49.80	49.60	48.94	50.91	45.94	48.27	54.35	53.46	49.82
F: Profit/loss per animal ** (ZAR)	−187.06	30.37	220.39	−237.83	719.23	423.39	−700.78	−494.41	251.35
G: Profit margin (%)	−1.45%	0.21%	1.42%	−1.68%	4.81%	2.62%	−4.69%	−2.97%	1.45%

A = Live entry weight ** ZAR 33.25/kg + 11.75% interest over the feeding period of 142 days (Red Meat Industry Services https://rmis.co.za/prices/; South African Reserve Bank https://www.resbank.co.za/en/home/what-we-do/statistics/key-statistics/selected-historical-rates (accessed on 3 March 2025)); B = (Mean daily feed intake (kg) * Feed cost/kg) + 50% [14]; C = A + B; D = Carcass weight (kg) * R55.00/kg (Reference: Red Meat Industry Services https://rmis.co.za/prices/ accessed on 3 March 2025); E = B ÷ Carcass gain (kg) over the feeding period (Table 1); F = D − C; G = E ÷ C (%). * Production Profile (PP) 3 represents feeder calves with low production potential based on phenotype. Production Profile (PP) 2 is a combination of PP 2+ and PP 2− data, representing all feeder calves with average production potential. Production Profile (PP) 1 represents feeder calves with high production potential based on phenotype. ** ZAR = South African Rand.

## Data Availability

The original contributions in the study are included in the article. Further enquiries can be directed to the corresponding author.

## References

[1] Department of Agriculture & Rural Development, 2005a. Feedlotting Cattle. https://www.kzndard.gov.za/images/Documents/RESOURCE_CENTRE/GUIDELINE_DOCUMENTS/PRODUCTION_GUIDELINES/Beef_Production/Feedlotting%20Cattle.pdf.

[2] AgriSETA_2018-_19-Annual_Report. www.agriseta.co.za.

[3] Red Meat Marketing, 2000; Department of Agriculture, 2003. https://www.nda.gov.za/phocadownloadpap/General_Publications/Agricultural%20Marketing%20Extension%20Training%20Paper%20No.7%20Livestock.pdf.

[4] Ford D., Leeuw K.L. (2002). South African feedlot industry and economics of beef production. The South African feedlot association. Chapter 2. Feedlot Management.

[5] Strydom P.E., Frylinck L., Montgomery J.L., Smith M.F. (2009). The comparison of three β-agonists for growth performance, carcass characteristics and meat quality of feedlot cattle. Meat Sci..

[6] Delmore R.J., Hodgen J.M., Johnson B.J. (2010). Perspectives on the application of zilpaterol hydrochloride in the United States beef industry. J. Anim. Sci..

[7] Agbeniga B., Webb E.C. (2018). Influence of carcass weight on meat quality of commercial feedlot steers with similar feedlot, slaughter and post-mortem management. Food Res. Int..

[8] Lombard W.A., Maré F.A., Jordaan H. (2018). The influence of animal traits on feedlot profitability of Santa Gertrudis cattle in South Africa. Agrekon.

[9] BFAP (2024). Baseline Outlook for South African Agricultural 2024–2033.

[10] Koknaroglu H., Loy D.D., Wilson D.E., Hoffman M.P., Lawerance J.D. (2005). Factors affecting beef cattle performance and profitability. Prof. Anim. Sci..

[11] Sy H.A., Faminow M.D., Johnson G., Crow G. (1997). Estimating the values of cattle characteristics using an ordered porbit model. Amer. J. Agr. Econ..

[12] Tatum J.D., Platter W.J., Bargen J.L., Endsley R.A. (2012). Carcass-based measures of cattle performance and feeding profitability. Prof. Anim. Sci..

[13] Retallick K.M., Faulkner D., Rodriguez-Zas S.L., Nkrumah J.D., Shike D.W. (2013). Relationship among performance, carcass, and feed efficiency characteristics, and their ability to predict economic value in the feedlot. J. Anim. Sci..

[14] Sartorello G.L., Bastras J.P., Gameiro A.H. (2018). Development of calculation model and production cost index for feedlot Beef cattle. Braz. J. Anim. Sci..

[15] Hoppe K. Extension Livestock Systems Specialist. Cattle Feeding Profits Depend on Input Costs, Rate of Gain. North Dakota State University..

[16] Batt R.A.L. (1980). Influences on Animal Growth and Development. The institute of biology’s studies in Biology No. 116.

[17] Hentzen A., Holm D.E. (2024). Novel production profile classification system for incoming calves that predicts feedlot growth performance. Anim. Prod. Sci..

[18] Zinn R.A., Barreras A., Owens F.N., Plascencia A. (2008). Performance by feedlot steers and heifers: Daily gain, mature body weight, dry matter intake, and dietary energetics. J. Anim. Sci..

[19] Braken Even Model Oklahoma State University. https://extension.okstate.edu/fact-sheets/program-to-estimate-feedlot-breakeven-purchase-price-beflcalc.html.

[20] Marques J.G.O., De OSilva R., Barioni L.G., Hall J.A.J., Tedeschi L.O., Moran D. (2020). An improved algorithm for solving profit-maximizing cattle diet problems. Animal.

[21] Hentzen A.H., Holm D.E. (2025). Precision Feeding of Feedlot Calves Based on Phenotypic Production Profiles I. The Effect on Economic Important Production Parameters. Animals.

[22] Tatum J.D., Williams F.L., Bowling R.A. (1986). Effects of feeder-cattle frame size and muscle thickness on subsequent growth and carcass development. I: An objective analysis of frame size and muscle thickness. J. Anim. Sci..

[23] Tatum J.D., Williams F.L., Bowling R.A. (1986). Effects of feeder-cattle frame size and muscle thickness on subsequent growth and carcass development. II: Absolute growth and associated changes in carcass composition. J. Anim. Sci..

[24] Hentzen A.H.R., Thompson P.N., Holm D.E. (2020). The effect of preconditioning on production and antibiotic use in a South African beef feedlot. Anim. Prod. Sci..

[25] Sturaro E., Quassolo M., Ramanzin M. (2005). Factors affecting growth performance in beef production: An on farm survey. Ital. J. Anim. Sci..

[26] Ryan W.J., Williams I.H., Moir R.J. (1993). Compensatory growth in sheep and cattle. 1. Growth pattern and feed intake. Aust. J. Agric. Res..

[27] Mukuahima G. (2007). The Performance of Beef Cattle Bulls in the Vrede District pf Mpumalanga. Master’s Thesis.

[28] Wells S. (2020). Prediction of the Growth Performance of Feedlot Cattle Using Phenotypic and Anthropometric Measures. Master’s Thesis.

[29] Reinhardt C.D., Busby W.D., Corah L.R. (2009). Relationship of various incoming cattle traits with feedlot performance and carcass traits. J. Anim. Sci..

[30] Ralston A.T., Davidson T.P., Kennick W.H. The Effect of Initial Weight, Time on Feed and pre Finishing Environment upon Feedlot Performance of Steers.

[31] Lamm D.W. Measuring Beef Cattle. Colorado State University Extension. https://mountainscholar.org/bitstream/handle/10217/182985/AEXTucsu206221809.pdf?Sequence=1&isAllowed=y.

[32] Hendrikson E.B., Kennedy D.W., Aiken G.E., Tabler S.F. (2005). Case Study: Assessment of relationships between carcass traits and body measure at conclusion of pasture backgrounding. Prof. Anim. Sci..

[33] Esterhuizen J., Groenewald I.B., Strydom P.E., Hugo A. (2008). The performance and meat quality of Bonsmara steers raised in a feedlot, on conventional pastures or on organic pastures. S. Afr. J. Anim. Sci..

[34] Lavarelli D., Bacnetti J., Guarino M. (2020). A review on dairy cattle farming: Is precision livestock farming the compromise for environmental, economic and social sustainable production?. J. Clean. Prod..

[35] Norris D., Macala Makore J., Mosimanyana B. (2002). Feedlot performance of various breed groups of cattle fed low to high levels of roughage. Livest. Res. Rural. Dev..

[36] Bosman D.J., Leeuw K.J. (2002). Cattle breeds and types for the feedlot. Agricultural research council, Animal Improvement institute, Irene In Chapter 6. Feedlot Management.

